# Methylome data derived from maternal-zygotic *DNA methyltransferase 3aa^−/−^* zebrafish

**DOI:** 10.1016/j.dib.2022.108514

**Published:** 2022-08-05

**Authors:** Masaki Shirai, Kazuya Takayama, Haruko Takahashi, Yudai Hirose, Masashi Fujii, Akinori Awazu, Nobuyoshi Shimoda, Yutaka Kikuchi

**Affiliations:** a Department of Biological Science, Graduate School of Science, Hiroshima University, Kagamiyama 1-3-1, Higashi-Hiroshima, Hiroshima, 739-8526 Japan; b Graduate School of Integrated Sciences for Life, Hiroshima University, Kagamiyama 1-3-1, Higashi-Hiroshima, Hiroshima 739-8526, Japan; c Department of Mathematical and Life Sciences, Graduate School of Science, Hiroshima University, Kagamiyama 1-3-1, Higashi-Hiroshima, Hiroshima, 739-8526 Japan; d Laboratory of Molecular Analysis, Research and Development Management Center, National Center for Geriatrics and Gerontology, 7-430, Morioka, Obu, Aichi 474-8522, Japan

**Keywords:** DNA methylation, Dnmt3aa, Zebrafish, Whole genome bisulfate sequencing

## Abstract

Genomic DNA methylation is an epigenetic marker mediated by DNA methyltransferases (Dnmts); in vertebrates, it comprises of a maintenance DNA methyltransferase, Dnmt1, and two *de novo* DNA methyltransferases (Dnmt3a and Dnmt3b). In zebrafish, there are two homologs of the mammalian Dnmt3a: Dnmt3aa and Dnmt3ab. A knockout (KO) mutant of zebrafish *dnmt3aa* was generated using the CRISPR/Cas9 genome-editing system as a new model for DNA methylation research. Since zebrafish *dnmt3aa* KO mutants were viable and fertile, a maternal-zygotic *dnmt3aa* deficient mutant (MZ*dnmt3aa*) was generated. We performed whole-genome bisulfite sequencing (WGBS) to reveal the DNA methylation profile using this mutant and identified genomic regions with altered CpG methylation as differentially methylated regions (DMRs) in this mutant compared to those in the wild-type fish. We provided novel raw and processed datasets using the MZ*dnmt3aa* KO mutant, and the raw data of WGBS are available through the Gene Expression Omnibus (GEO), accession number GSE178690.

## Specifications Table

**Table:** 

Subject	Biology
Specific subject area	Genetics, Genomics, Epigenetics
Type of data	Tables and Figures
How the data were acquired	WGBS libraries were obtained from bisulfite-treated genomic DNA from wild-type and MZ*dnmt3aa^−/−^* zebrafish. Sequencing was performed using the HiSeq X Five Sequencing System (Illumina) with 150 bp single-end reads.
Data format	Raw and analyzed
Description of data collection	Two pools, wild type (WT) and MZ*dnmt3aa^−/−^*, were prepared, each consisting of 10 larvae at 2 days post fertilization (dpf). Genomic DNA was then extracted by digesting each pool with proteinase K and sodium dodecyl sulfate. DNA samples were subjected to bisulfite treatment using the EZ DNA Methylation Gold Kit (Zymo Research). Adapters were added to the bisulfite-treated DNA samples by PCR using random primers from the TruSeq DNA Methylation Kit (Illumina). WGBS data were obtained from a single experiment.
Data source location	• *Institution:* Hiroshima University • *City/Town/Region:* Higashi-Hiroshima, Hiroshima • *Country:* Japan
Data accessibility	Repository name: Gene Expression Omnibus (GEO) Data identification number: accession number of SuperSeries is GSE178691. accession number of SubSeries is GSE178690. Direct URL to data: https://www.ncbi.nlm.nih.gov/geo/query/acc.cgi?acc=GSE178691 https://www.ncbi.nlm.nih.gov/geo/query/acc.cgi?acc=GSE178690
Related research article	Not Applicable

## Value of the Data

• A new epigenomic dataset is provided for the MZ*dnmt3aa^−/−^* mutant to study DNA methylation in zebrafish.
• Since there are two *dnmt3a* genes in zebrafish, *dnmt3aa* and *dnmt3ab*, the WGBS data for the zebrafish MZ*dnmt3aa^−/−^* mutant is useful for the detailed analysis of target genomic loci by Dnmt3a in vertebrates.
• Enables genome-wide assessment in zebrafish regarding Dnmt3aa-mediated changes in DNA methylation.
• Our dataset is useful for studies examining the effects of DNA methylation on transcription because hypomethylated differentially methylated regions (HypoDMRs) were able to be detected that overlap not only the transcription start sites and gene bodies, but also the transcription termination sites.

## 1 Data Description

**Fig. 1:**
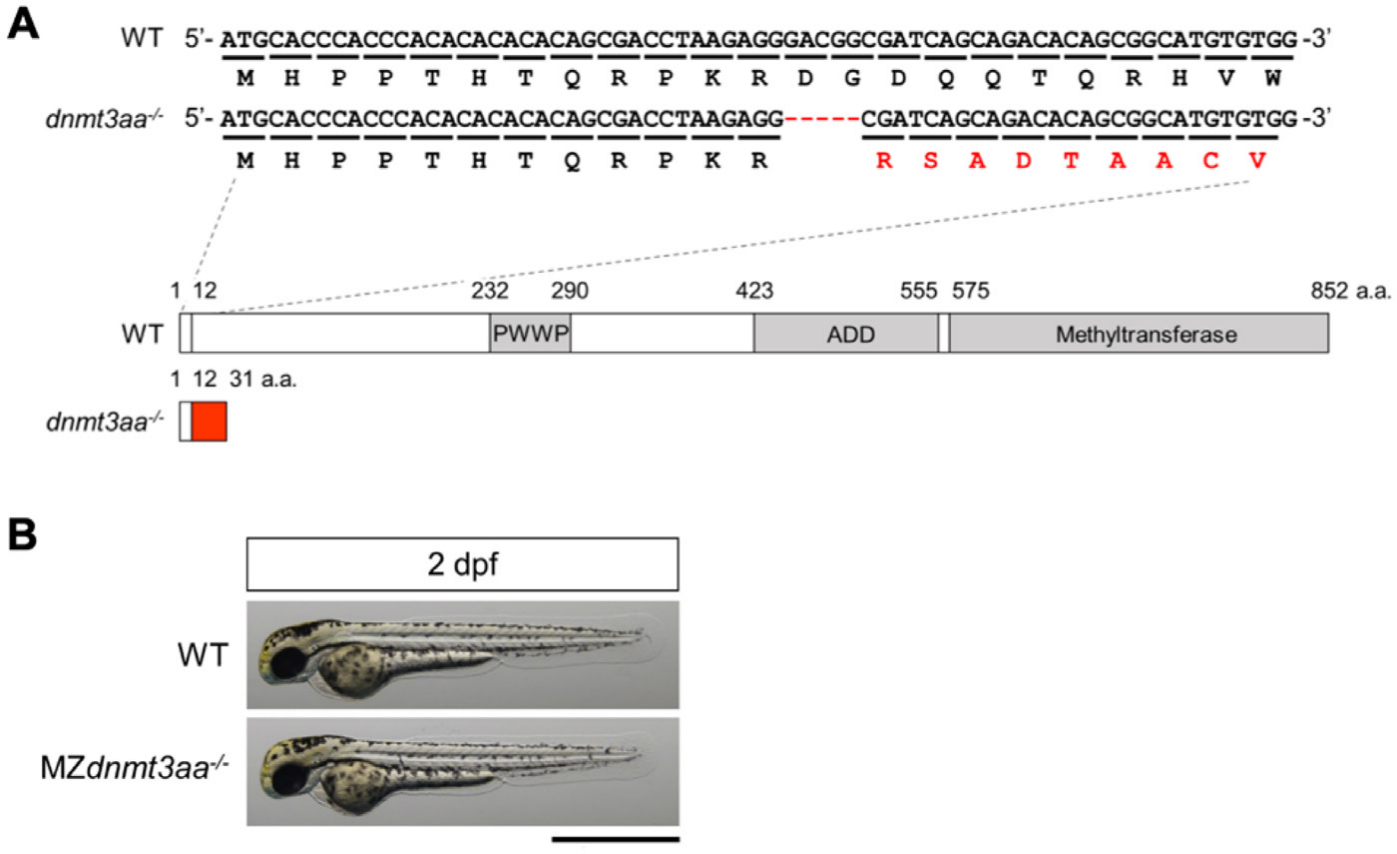
Generation of MZ*dnmt3aa*^-/-^ zebrafish mutant (A) The *dnmt3aa* genomic DNA sequence and CRISPR/Cas9-mediated indel mutation, with predicted amino acid sequences, in WT and MZ*dnmt3aa*^-/-^ mutants are shown. The MZ*dnmt3aa*^-/-^ mutants have a 5-bp deletion which leads to a frame-shift at amino acid 13^th^ position. The schematic diagram shows predicted protein products from the WT and the mutant alleles. It is speculated that the deletion mutation results in the production of a truncated protein with additional sequences (red box) at the C-termini. Putative functional domains (PWWP, cysteine-rich ATRX-Dnmt3-Dnmt3L (ADD), and methyltransferase domains) in the WT protein are represented as grey colored boxes. (B) Gross morphology of the MZ*dnmt3aa*^-/-^ mutant is identical to WT at 2 dpf. Scale bar: 1 mm.

Here, we present WGBS data for WT and MZ*dnmt3aa^−/−^* zebrafish. A deletion of *dnmt3aa* gene was generated using CRISPR/Cas9. The *dnmt3aa* genomic DNA sequence and CRISPR/Cas9-mediated indel mutation, with the predicted amino acid sequences, in the WT and MZ*dnmt3aa^−/−^* mutant are shown in Fig. 1. Fig. 1 is a modified version from a larger Fig. published in Shirai et al. bioRxiv. (2021) [1].

To investigate the DNA methylation profile changed by *dnmt3aa* knockout, we performed WGBS and identified hypoDMRs. The data is as follows:

Supplementary Table S1: HypoDMRs overlapping transcription start sites (TSSs)

Supplementary Table S2: HypoDMRs in the gene bodies

Supplementary Table S3: HypoDMRs overlapping transcription termination sites (TTSs)

Supplementary Table S4: HypoDMRs in the intergenic regions

Supplementary Table S5: Genes with hypoDMRs overlapping TSSs

Supplementary Table S6: Genes with hypoDMRs in the gene bodies

Supplementary Table S7: Genes with hypoDMRs overlapping TTSs

WGBS data are deposited in the GEO under accession numbers GSE178691 and GSE178690.

## 2 Experimental Design, Materials and Methods

### 2.1 Zebrafish experiments and generation of *dnmt3aa^−/−^* mutant fish using CRISPR/Cas9 system

Adult zebrafish and zebrafish larvae were maintained as described previously [2]. Genome editing using CRISPR/Cas9 system was performed as previously described [3]. Briefly, the pDR274 vector (Addgene Plasmid 42250) was digested with *Bsa*I, and annealed oligonucleotides for sgRNAs were cloned into the pDR274 vector. The sequences of oligonucleotides for sgRNAs are listed in Table 1.

**Table 1:** Oligonucleotide sequences for the construction of sgRNAs.

sgRNA ID	oligonucleotide sequence (5′ to 3′)	gRNA target site (5′ to 3′)
No.1	TAGGACCCACACACACACAGCG	GTCGCTGTGTGTGTGTGGGTGGG
	AAACCGCTGTGTGTGTGTGGGT	
No.2	TAGGACAGCGACCTAAGAGGGA	ACACAGCGACCTAAGAGGGACGG
	AAACTCCCTCTTAGGTCGCTGT	
No.3	TAGGTTGGATATGGCATTGGAG	GCTCTCCAATGCCATATCCAAGG
	AAACCTCCAATGCCATATCCAA	

The sgRNA expression vectors were digested with *Dra*I, and the sgRNAs were synthesized using the MAXIscript™ T7 Kit (Invitrogen, Thermo Fisher Scientific). The synthesized sgRNAs were purified using the mirVana™ miRNA Isolation Kit (Invitrogen, Thermo Fisher Scientific). Moreover, the Cas9 expression vector (Addgene Plasmid 51815) was linearized using *Not*I, and *Cas9* mRNA was synthesized using the mMESSAGE mMACHINE™ SP6 Kit (Invitrogen, Thermo Fisher Scientific). A mixture of three types of sgRNAs for the zebrafish *dnmt3aa* gene and *Cas9* mRNA at appropriate concentrations was injected at the one-cell stage.

To identify the mutations, the target site of the CRISPR/Cas9 system on *dnmt3aa* was amplified using TaKaRa Ex Taq® (Takara Bio) with the primers listed in Table 2. The amplicons were purified using the QIAquick PCR Purification Kit (QIAGEN) and then sequenced using the BigDye™ Terminator v3.1 Cycle Sequencing Kit (Applied Biosystems, Thermo Fisher Scientific) and the primers listed in Table 2.

**Table 2:** Primer sequences to confirm *dnmt3aa* mutation.

primer sequence (5′ to 3′)	objective
Forward: GGTGACTTCACGGATACTACGTC	target amplification for sequencing
Reverse: CACATACCCACACATGCCGCTGT	
GTTCAGAGCTGTACGTCTTCCTG	sequencing
Forward: TCAGCATCGAGTCATGTGATGCC	genotyping (HMA assay)
Reverse: CACATACCCACACATGCCGCTGT	

### 2.2 Genotyping of MZ*dnmt3aa^−/−^* zebrafish

The amputated fins of MZ*dnmt3aa^−/−^* mutants were digested overnight in lysis buffer (100 mM NaCl, 20 mM Tris-HCl pH 8, 50 mM EDTA pH 8, 0.5% SDS, and 36 ug/ml Proteinase K) at 55°C. Proteinase K was inactivated by incubation at 98°C for 10 min. A heteroduplex mobility assay (HMA) was performed to detect the *dnmt3aa* mutations. The target site of the CRISPR/Cas9 system on exon 1 of the *dnmt3aa* gene was amplified using BIOTAQ™ DNA Polymerase (Meridian Bioscience). The primers used are listed in Table 2. The amplicons were analyzed using polyacrylamide gel electrophoresis.

### 2.3 Whole-genome bisulfite library preparation and sequencing of zebrafish sample

Two pools, WT and MZ*dnmt3aa^−/−^*, were prepared, each consisting of 10 larvae at 2 dpf. Genomic DNA was then extracted by digesting each pool with proteinase K and sodium dodecyl sulfate. DNA samples were prepared by mixing zebrafish genomic DNA and unmethylated lambda DNA (Promega) as spike controls at a ratio of 1000:1. DNA samples (100 ng) were subjected to bisulfite treatment using an EZ DNA Methylation-Gold Kit (Zymo Research). Bisulfite-treated DNA samples (50 ng) were subjected to ten cycles of PCR using random primers from the TruSeq DNA Methylation Kit (Illumina) to add adapters. Libraries with adapters were sequenced on a HiSeq X Five Sequencing System (Illumina) with 150 bp single-end reads. WGBS data were obtained from a single experiment. Bisulfite treatment, library preparation, and sequencing were performed by Takara Bio, Inc. (Shiga, Japan).

### 2.4 WGBS data processing of zebrafish sample

The read sequences in FASTQ format were trimmed by removing six bases from the random primers of the TruSeq DNA Methylation Kit (Illumina) and the last 50 bases of low-quality reads. After trimming, the reads were aligned to the zebrafish (danRer10) and lambda phage genomes using Bismark (version 0.10.1) [4] and Bowtie (version 1.0.0) [5]. Once aligned, reads corresponding to PCR duplicates were removed and methylation calls were performed. The bisulfite conversion rates based on spike control for all samples were over 99%, and the error rates were as follows: WT, 0.3% and MZ*dnmt3aa^−/−^* mutant, 0.3%. Methylated cytosine sites were identified using binomial distribution with a false discovery rate ≤ 0.05, calculated using the Benjamini-Hochberg (BH) method and read depth ≥ 5. Data analysis was performed using Takara Bio, Inc. (Shiga, Japan), as previously described [6].

### 2.5 Identification of differentially methylated regions (DMRs)

Methylation call data were used to identify DMRs using swDMR (version 1.0.0) [7] with the following settings: cytosine type, CG; window, 500; step size, 50; points (lowest number of selected cytosine types in the window), 3; coverage, 5; fold (methylation level difference), 1.5; diff (value of max-min methylation level), 0.1; p-value, 0.05; fdr, 0.05. The samples were compared using Fisher's test. HypoDMRs were identified from the DMRs data.

### 2.6 Identification of HypoDMRs with TTSs, TSSs, gene bodies, and intergenic regions

The genomic positions of HypoDMRs overlapping with TTSs, TSSs, gene bodies, and intergenic regions were determined using bedtools intersect (version 2.28.0) [8]. HypoDMRs that overlapped with TTSs and the other three (TSSs, gene bodies, and intergenic regions) were included in the HypoDMRs that overlapped with TTSs. HypoDMRs that overlapped with TSSs and the other two (gene bodies and intergenic regions) were included in HypoDMRs that overlapped with TSSs. HypoDMRs that overlapped with both gene bodies and intergenic regions were included in the HypoDMRs that overlapped with gene bodies. HypoDMRs that did not overlap with TTSs, TSSs, or gene bodies were included in the HypoDMRs that overlapped with an intergenic region. In addition, genes with the HypoDMRs were extracted concurrently.

## Ethics Statements

All zebrafish experiments were carried out with approval from the Hiroshima University Animal Research Committee (Permit Number: F18-2-7).

## CRediT authorship contribution statement

**Masaki Shirai:** Investigation, Data curation, Validation, Formal analysis, Writing – original draft, Writing – review & editing, Visualization. **Kazuya Takayama:** Resources, Investigation. **Haruko Takahashi:** Supervision, Investigation, Writing – review & editing. **Yudai Hirose:** Data curation, Formal analysis. **Masashi Fujii:** Supervision, Data curation. **Akinori Awazu:** Supervision, Data curation. **Nobuyoshi Shimoda:** Conceptualization, Funding acquisition, Writing – review & editing. **Yutaka Kikuchi:** Conceptualization, Supervision, Writing – original draft, Writing – review & editing, Funding acquisition, Project administration.

## Declaration of Competing Interest

The authors declare that they have no known competing financial interests or personal relationships that could have appeared to influence the work reported in this paper.

## Data Availability

Identification of transcription termination defects at DNA hypomethylated transcription termination sites in DNA methyltransferase 3a-deficient vertebrates [WGBS] (Original data) (NCBI Gene Expression Omnibus (GEO) repository under GEO accession GSE178690)

## Appendix Supplementary materials

Image, application 1

Image, application 2

Image, application 3

Image, application 4

Image, application 5

Image, application 6

Image, application 7
